# Supplementary figures and images for: Factors Influencing Public Willingness to Reuse the Unused Stored Medications in Jordan: A Cross-Sectional Study
Source: Healthcare (Basel). 2022 Dec 27;11(1):75. doi: 10.3390/healthcare11010075 (PMC9818750; doi:10.3390/healthcare11010075)

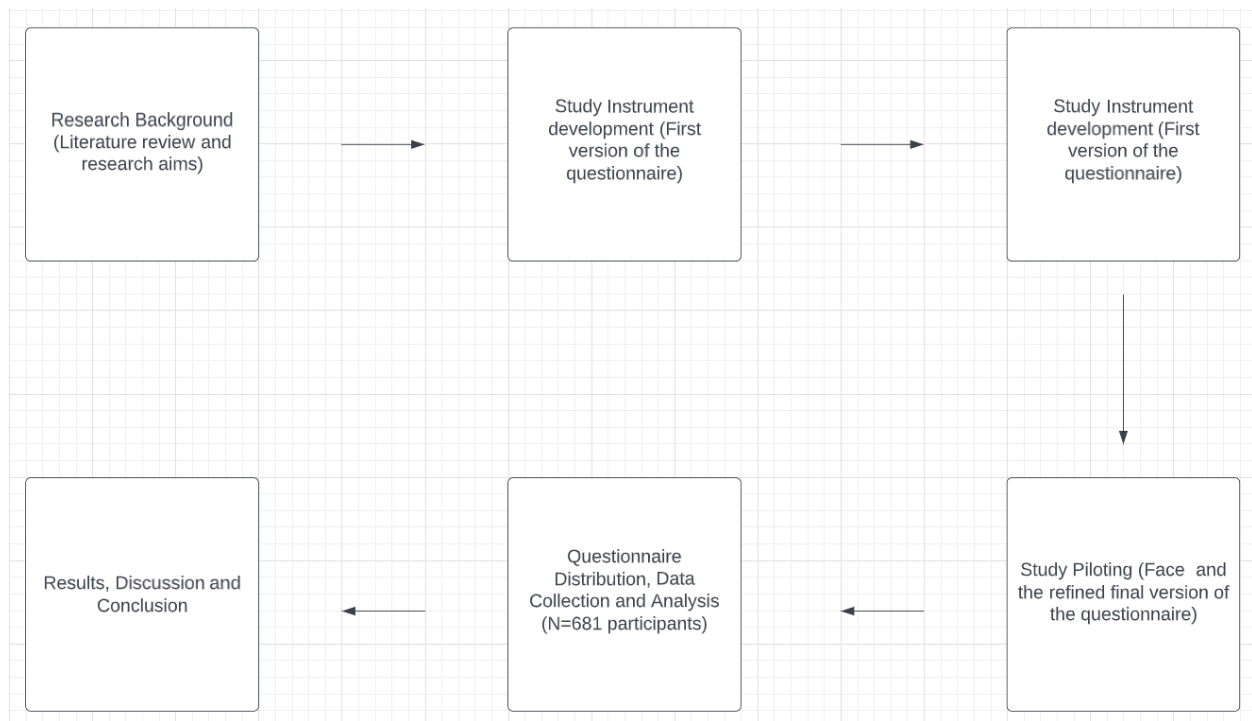

Figure S1. Flow chart of the whole study process

Supplement: Supplementary file 1 [file healthcare-11-00075-s001.zip › healthcare-2092711-supplementary.pdf]
